# Supplementary material for: Polysaccharide Coating of Gelatin Gels for Controlled BSA Release
Source: Polymers (Basel). 2019 Apr 17;11(4):702. doi: 10.3390/polym11040702 (PMC6523836; doi:10.3390/polym11040702)
Supplement: Supplementary file 1 [file polymers-11-00702-s001.pdf]

SUPPORTING INFORMATION

Nanocoating of Gelatin Gels for Controlled BSA Release

Jimena S. Gonzalez[12*], Carmen Mijangos[2] and Rebeca Hernandez[2]

[1] Institute of Materials Science and Technology (INTEMA), University of Mar del Plata and National Research Council (CONICET), Colón 10890, 7600 Mar del Plata, Argentine.
[2] Instituto de Ciencia y Tecnología de Polímeros (ICTP), CSIC, Juan de la Cierva 3, Madrid, Spain
* Correspondence: Jimena S. Gonzalez. jimena.gonzalez@fi.mdp.edu.ar. Institute of Materials Science and Technology (INTEMA), University of Mar del Plata and National Research Council (CONICET), Colón 10890, 7600 Mar del Plata, Argentina. TE: +54 223 6260600 ext. 1470
Received: date; Accepted: date; Published: date

S1. Precipitation studies

A series of precipitation studies were carried out in order to determine the best conditions for the interaction of gelatin with both sprayed polymers. pH and concentration of Alginate and Chitosan solutions were selected from a previous work. For the precipitation study, 1mL of each solution of Alg (2.5mg/mL, pH=3) and Chi (1mg/mL, pH=5) were mixed with gelatin solutions at pH=3 and pH=5.

Figure S1 shows all the tested combinations. As a first observation all samples can form gels. In spite of the all samples did not show precipitated, alginate with gelatin gels (fig.S1a-c) has turbidity indicanding weak interactions in all pH tested. In the case of chitosan with gelatin (figS1d-f) transparent gels were obtained. Considering these results, the sandwich samples were prepared attempting the gelatin gels were in contact with the alginate.

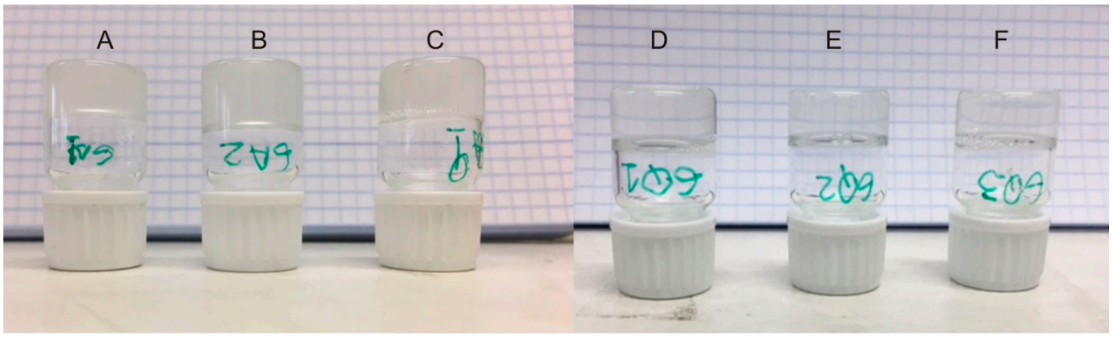

Figure S1. photographs of interactions studies, a)-c) 5mg/mL Alginate solution, pH=3 with : a) Gelatin solution 10%w/v, pH=3 b) Gelatin solution 10%w/v, pH=5 c) Gelatin solution 10%w/v as prepared d)-f) 1mg/mL Chitosan solution, pH=5 with d) Gelatin solution 10%w/v, pH=3 e) Gelatin solution 10%w/v, pH=5 f) Gelatin solution 10%w/v as prepared.

S2. Raman Spectroscopy

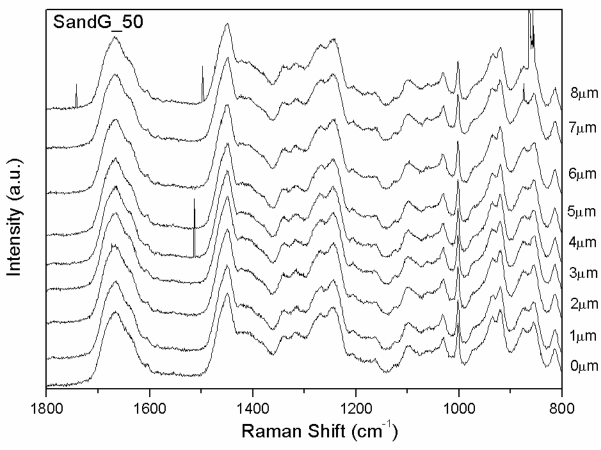

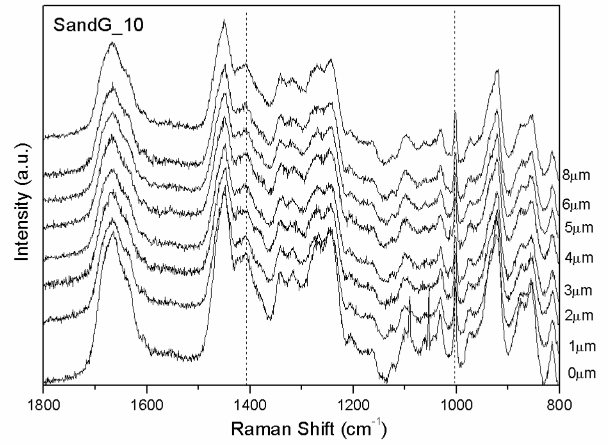

Figure S2.(a) Depth profile of normalized Raman spectra corresponding to the sample SandG_50 and *(b)* Depth profile of normalized Raman spectra corresponding to the sample SandG_10
